# A glycosylated recombinant human granulocyte colony stimulating factor produced in a novel protein production system (AVI-014) in healthy subjects: a first-in human, single dose, controlled study

**DOI:** 10.1186/1472-6904-9-2

**Published:** 2009-01-28

**Authors:** Roslyn Varki, Ed Pequignot, Mark C Leavitt, Andres Ferber, Walter K Kraft

**Affiliations:** 1 Department of Pharmacology and Experimental Therapeutics, Thomas Jefferson University, 132 South 10th Street, 1170 Main Building, Philadelphia, PA 19107, USA; 2 Synageva Biopharma, 111 Riverbend Road, Athens, GA 30605, USA; 3 Cancer Institute of New Jersey at Cooper University Hospital, Camden, NJ 08103, USA

## Abstract

**Background:**

AVI-014 is an egg white-derived, recombinant, human granulocyte colony-stimulating factor (G-CSF). This healthy volunteer study is the first human investigation of AVI-014.

**Methods:**

24 male and female subjects received a single subcutaneous injection of AVI-014 at 4 or 8 mcg/kg. 16 control subjects received 4 or 8 mcg/kg of filgrastim (Neupogen, Amgen) in a partially blinded, parallel fashion.

**Results:**

The Geometric Mean Ratio (GMR) (90% CI) of 4 mcg/kg AVI-014/filgrastim AUC(0–72 hr) was 1.00 (0.76, 1.31) and Cmax was 0.86 (0.66, 1.13). At the 8 mcg/kg dose, the AUC(0–72) GMR was 0.89 (0.69, 1.14) and Cmax was 0.76 (0.58, 0.98). A priori pharmacokinetic bioequivalence was defined as the 90% CI of the GMR bounded by 0.8–1.25. Both the white blood cell and absolute neutrophil count area under the % increase curve AUC(0–9 days) and Cmax (maximal % increase from baseline)GMR at 4 and 8 mcg/kg fell within the 0.5–2.0 a priori bound set for pharmacodynamic bioequivalence. The CD 34+ % increase curve AUC(0–9 days) and Cmax GMR for both doses was ~1, but 90% confidence intervals were large due to inherent variance, and this measure did not meet pharmacodynamic bioequivalence. AVI-014 demonstrated a side effect profile similar to that of filgrastim.

**Conclusion:**

AVI-014 has safety, pharmacokinetic, and pharmacodynamic properties comparable to filgrastim at an equal dose in healthy volunteers. These findings support further investigation in AVI-014.

## Background

Granulocyte colony stimulating factor (G-CSF) is a cytokine produced by monocytes, macrophages, endothelial cells, and fibroblasts. Human G-CSF consists of 174 amino acids with an approximate molecular weight of 20 kDa. The native protein is O-glycosylated on threonine 133. G-CSF plays a critical role in the modulation of neutrophil biology. It is required to maintain adequate basal neutrophil count, as well as generation of an appropriate neutrophilia in response to infectious stimuli [1]. Primary effects of G-CSF on neutrophils include an increase in cell division and a decrease in marrow transit time; the net effects of both functions being to increase the total neutrophils [2]. Other effects of G-CSF on neutrophils include attraction and localization to sites of infection, increase in phagocytosis and a decrease in apoptosis.

Filgrastim is a recombinant human granulocyte colony stimulating factor (rhG-CSF) which was developed in the mid-1980's and FDA-approved for use in chemotherapy induced neutropenia in 1991 [3]. Filgrastim is a non-glycosylated protein produced in *E. coli *bacteria transfected with rhG-CSF cDNA. Two other products approved in several countries include lenograstim, which is derived from mammalian cells and is glycosylated, and nartograstim which is non-glycosylated and has 5 different amino acids at the N-terminal region as compared with the natural human G-CSF. All the three versions of rhG-CSF have been successfully used to treat chemotherapy-induced or other forms of neutropenia for over a decade and appear to have similar efficacy [4]. Specific indications for rhG-CSF include primary prophylaxis in high-risk patients receiving chemotherapy, treatment of chemotherapy-induced neutropenic fever, and use in hematopoietic stem cell transplantation [5]. In 2002, a pegylated filgrastim with extended duration of action relative to the naked filgrastim was approved by the FDA.

AVI-014 is a transgenic glycosylated rhG-CSF produced by purification from egg whites of hens transfected with hG-CSF cDNA. The threonine residue at position 133 is glycosylated and the protein has a molecular weight of approximately 20 kD. Matrix assisted laser desorption/ionization time-of-flight (MALDI-TOF) analysis shows this species has the same polypeptide sequence as the natural human G-CSF. AVI-014 shows immunoreactivity with rhG-CSF-specific antibodies in a Western blot assay and bioactivity in a cell proliferation assay. Biologic activity (~1.5 × 10^8 ^IU/mg) in NFS-60 cells shows equipotency to filgrastim.

The goal of this first-in-human study was to characterize the safety, tolerability, pharmacokinetics, and pharmacodynamics of AVI-014 in healthy human volunteers upon subcutaneous administration of a single dose, and to compare it to the existing commercially available rhG-CSF product, filgrastim.

## Methods

### Subjects

Forty healthy volunteers were enrolled in this study. Written informed consent was obtained from each subject prior to the initiation of any study procedures. The study protocol conformed to the ethical guidelines of the Declaration of Helsinki as reflected in approval by the Institutional Review Board of Thomas Jefferson University. Subjects were considered to be in good health on the basis of medical history, physical examination, laboratory values, and electrocardiograms (ECG). No subject used prescription or over-the-counter medications, with the exception of hormonal contraceptives, within the period starting 14 days prior to first administration of the study drug. Allergy to chicken eggs or influenza vaccine was an exclusion criteria. Subjects with a body mass index between 19 and 30 were enrolled

### Study design

This was a single escalating dose study comprised of three treatment groups. Eight subjects in Panel A received an open label, single subcutaneous dose of AVI-014 at a dose of 4 μg/kg. Subjects in Panel B were administered with either a single subcutaneous dose of AVI-014 4 μg/kg (n = 8) or an equivalent dose of filgrastim (Neupogen, Amgen) 4 μg/kg (n = 8) in a double blinded fashion. Subjects in Panel C were administered with either a single subcutaneous dose of AVI-014 8 μg/kg (n = 8) or an equivalent dose of filgrastim 8 μg/kg (n = 8) in a double blinded fashion. This was a first-in-human administration of AVI-014, and no formal power calculation was performed. Prior to unblinding, the computer-generated randomization scheme was accessible only to the study statistician and to the unblinded study staff member. Neither of these individuals assessed adverse events. All pharmacokinetic and pharmacodynamic analysis was performed prior to unblinding.

Each subject received a comprehensive medical history and inquiry into concomitant medication use, complete physical examination, clinical laboratory safety assessments (hematology, blood chemistry, liver function tests, and urinalysis), and ECG during screening. Females had a negative serum pregnancy test during screening as well as a negative urine pregnancy test on the first day of the study prior to study drug administration. Subjects received a single subcutaneous injection in the abdomen of study drug followed by pharmacokinetic and pharmacodynamic sampling. ECG examination was performed at screening, within 1 hour prior to dosing, and at 6 hours and 21 days post study drug administration. Injection sites were monitored for signs of irritation or inflammation.

### Pharmacokinetics

Blood for pharmacokinetics was drawn at baseline and 0.5, 1, 2, 3, 4, 5, 6, 7, 8, 10, 12, 24, 48, and 72 hours after drug administration. Blood samples were allowed to clot and serum was collected and stored at <-20°C. Serum levels of AVI-014 and control filgrastim were determined using a hG-CSF specific ELISA kit (R&D Systems, Minneapolis, MN). Concentration was calculated by interpolation using a standard curve generated with either AVI-014 or filgrastim. The limit of quantification of the assay was 7.8 pg/ml.

### Pharmacodynamics

Pharmacodynamic endpoints included absolute neutrophil count (ANC), total white blood cell (WBC) count and total CD 34+ cell count. A complete blood count was obtained predose and on days 2–9. Blood for flow cytometry was collected predose and days 2–6. With the exception of the predose draw, which was collected at approximately 20:00 on day -1, blood for pharmacodynamic evaluation was collected at the dose time. CD 34+ counts were performed by the Thomas Jefferson University Hospital Clinical Laboratory. During flow cytometry of subject samples, the gated area for CD 34 positive cells was determined by visual inspection with an effort to maintain consistency between samples. All flow cytometry and clinical laboratory personnel were blinded during the conduct of the study.

### Pharmacokinetic data analysis

Pharmacokinetic analysis was performed using WinNonLin Enterprise version 5.1 (Pharsight Inc., Mountain View, CA). A non-compartmental approach was employed. Area under the curve (AUC) was determined using the linear/log trapezoidal rule. Actual sample collection times were used for the generation of pharmacokinetic parameters. Individual time concentration plots were examined for each subject. Appropriateness of time points to be used in calculation of the elimination rate constant was verified by visual examination of the terminal time points. In all cases the terminal elimination was determined from the 24 to 72 hour time point. Estimation of an alpha half life was performed using the time points of 8 to 24 hours.

### Statistical methods

#### Pharmacokinetics

AUC's and Cmax values were natural log-transformed. Means, standard deviations, min, max and medians were calculated for each dose level and group (AVI-014 and filgrastim). Arithmetic means were be back-transformed (by taking anti-logs) to obtain geometric means. The a priori bounds that would define pharmacokinetic equivalence of AVI-014 and filgrastim were a 90% confidence interval of geometric mean ratio of area under the time concentration curve (AUC(0–72)) and of the maximal G-CSF concentration (Cmax) that lies between 0.8 and 1.25. A linear mixed effects model was used to compare AVI-014 and filgrastim for the parameters of AUC(0–72) and Cmax. This model had treatment as a fixed effect. Panel and session nested within panel were also fixed effects. The variances were calculated for each group, using subject as random effects. Ratios (AVI-014/filgrastim) of geometric means were calculated by back-transforming the difference of least-squares mean, and a 90% confidence interval calculated for this ratio. Residuals were computed and examined for normality.

#### Pharmacodynamics

WBC, ANC and CD 34+ were analyzed as percentage increase from the baseline, with determination of an area under the % increase curve (AUC(0–9 day) for WBC and ANC, and AUC(0–6 day) for CD34+), the maximal % increase from baseline (Cmax). Units for AUC are % increase-day; and for Cmax are % increase. The distance between day 0 and day 2 was considered to be 1.5 days for the purposes of determination of area under the curve. Geometric mean ratios of AUC and Cmax were compared, and the data were analyzed in the same fixed effects model as for pharmacokinetics. The a priori bounds that defined pharmacodynamic equivalence of AVI-014 and filgrastim were a 90% confidence interval of geometric mean ratio of area under the % increase curve (AUC) and of the maximal % increase from baseline (Cmax) that lay between 0.5 and 2.0.

## Results

### Subjects

The mean age of the subjects enrolled in the trial was 34.5 years (range 21–50). Twenty nine (73%) of the subjects were male. Thirteen (32.5%) of the subjects were white and 27 (67.5%) were black. The mean weight was 81 kg (range 61–106). Demographics by treatment allocation are presented in Table 1. All subjects who were allocated to treatment received study drug (AVI-014 or filgrastim) and all 40 subjects who enrolled completed the study.

**Table 1:** Subject demographics by treatment allocation

	**Panel A**		**Panel B**		**Panel C**	
	**Category**	**AVI-014 4 μg/kg**	**AVI-014 4 μg/kg**	**Filgrastim 4 μg/kg**	**AVI-014 8 μg/kg**	**Filgrastim 8 μg/kg**

	n	8	8	8	8	8

Race n (%)	White	4 (50.0)	2 (25.0)	3 (37.5)	3 (37.5)	1 (12.5)
	
	Black	4 (50.0)	6 (75.0)	5 (62.5)	5 (62.5)	7 (87.5)

Gender n (%)	Male	7 (87.5)	5 (62.5)	4 (50.0)	5 (62.5)	8 (100.0)
	
	Female	1 (12.5)	3 (37.5)	4 (50.0)	3 (37.5)	0 (0.0)

Age (years)	Mean (SD)	33.6 (7.8)	35.4 (6.6)	30.3 (5.1)	33.8 (5.7)	39.6 (7.3)

Weight (kg)	Mean (SD)	78.3 (10.4)	81.2 (12.1)	79.4 (7.9)	76.3 (6.1)	90.6 (11.9)

Height (cm)	Mean (SD)	177 (11)	175 (8)	170 (10)	173 (5)	181 (4)

### Pharmacokinetics

**Figure 1:**
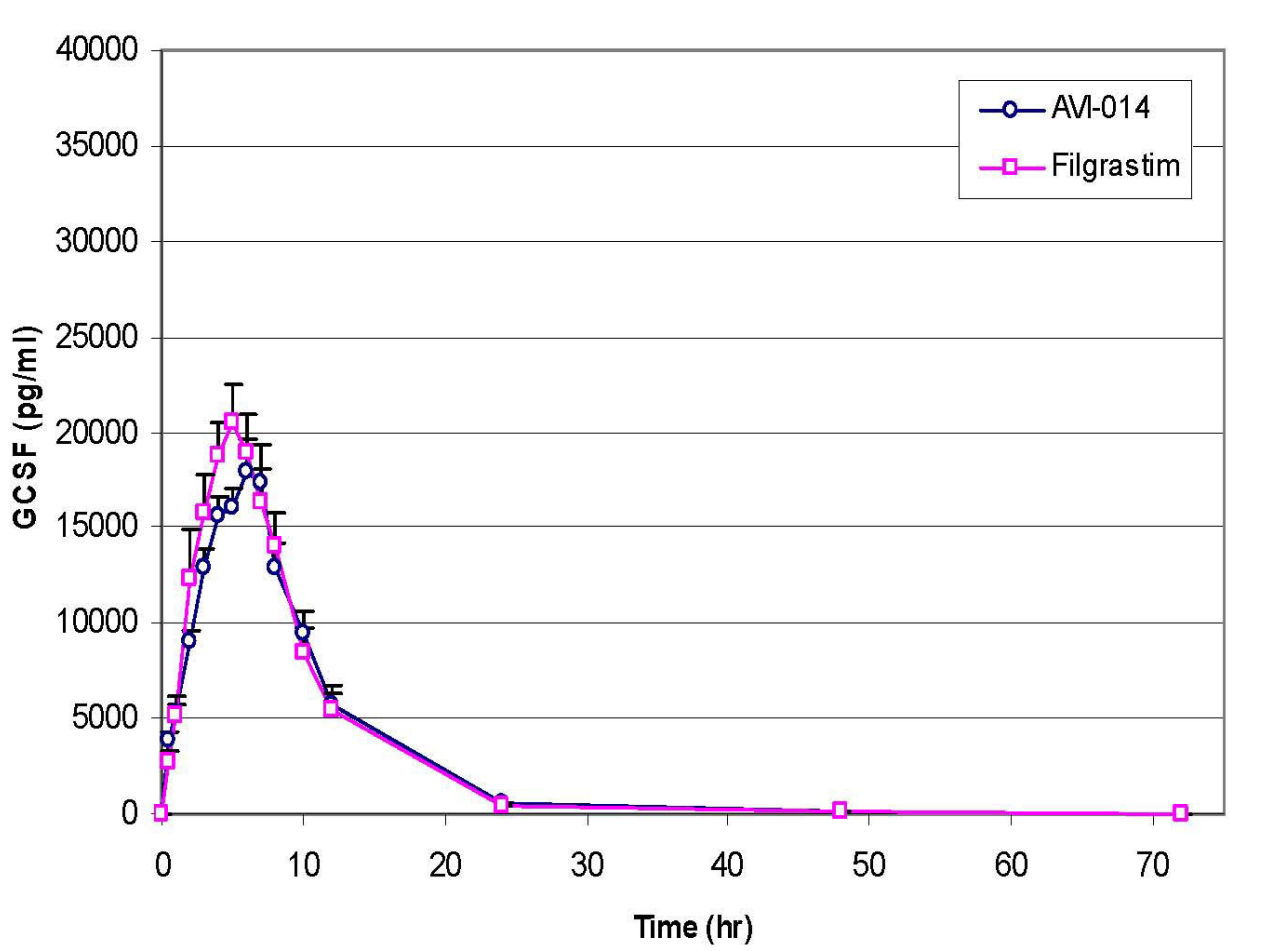
**4 μg/kg Dose of AVI-014 and Filgrastim, (with standard error)**.

**Figure 2:**
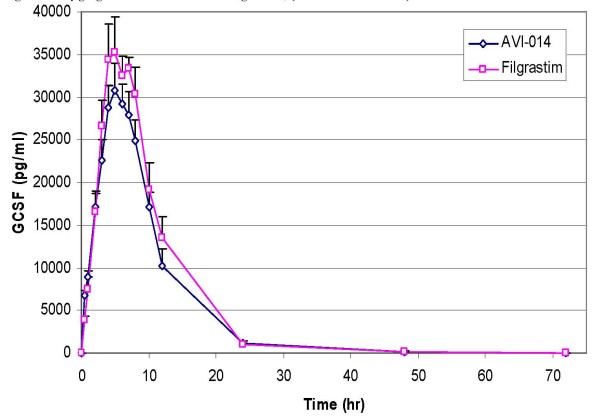
**8 μg/kg Dose of AVI-014 and Filgrastim, (with standard error)**.

**Figure 3:**
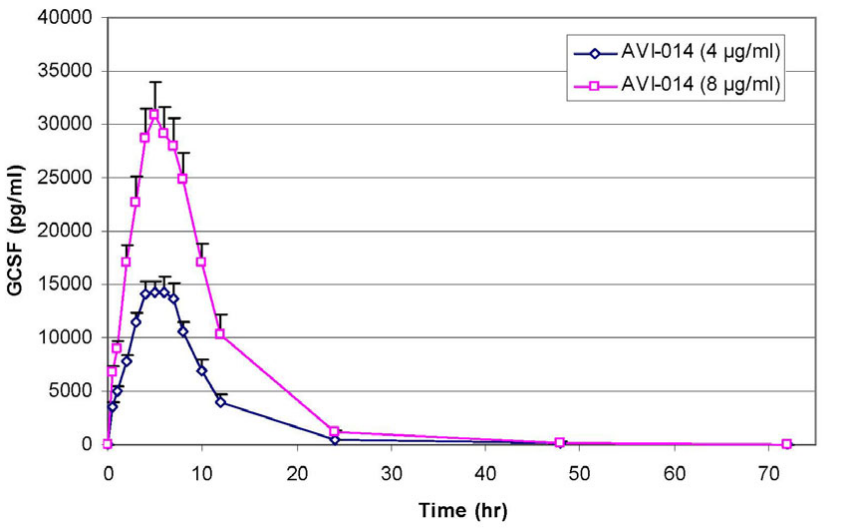
**AVI-014 at 4 μg/kg and 8 μg/kg (with standard error)**.

A summary of PK parameters by treatment for all groups is presented in Table 2. Graphical representation of concentration vs. time plots are presented in Figures 1, 2 (between treatments at the same dose level) and in Figure 3 (between doses for the same treatment). A summary of geometric mean ratios comparing AVI-014 and filgrastim at equal doses are presented in Tables 3 (4 μg/kg, Panel B only) and Table 4 (8 μg/kg). At the 4 μg/kg dose level, AVI-014 and filgrastim had similar AUC(0–72) values as evidenced by a point estimate of the geometric mean ratio of 1.00. However, the 90% confidence interval is outside the a priori bioequivalence bounds of 0.8–1.25. The point estimates for Cmax for the 4 μg/kg range, as well as AUC(0–72) and Cmax for the 8 μg/kg dose show slightly higher values for filgrastim. There was no formal hypothesis testing examining other pharmacokinetic parameters, but at equivalent mass doses, AVI-014 and filgrastim appear generally comparable in terms of clearance, volume of distribution, terminal elimination half life, and the estimated alpha half life generated by examining the slope of the exponential decline between time points 8 and 24 hours. The geometric mean ratios between treatments did not appreciably change when analysis was performed using combined data from Panels A and B compared to using only blinded administration in Panel B. Comparisons presented here employ blinded administration data (i.e., Panel B only).

**Table 2:** Pharmacokinetic parameters by treatment and dose level

Treatment	Dose	N	Cmax	AUC_(0–72)_	AUC_(0-∞)_	Clearance	Terminal elimination half life	Estimated Alpha half life	Volume of distribution	Volume of distribution	Tmax*
	mcg/kg		pg/mL	hr*pg/mL	hr*pg/mL	mL/min	hr	hr	L	L/kg	hr

AVI-014	4	16	16,944 *(5,804)*	138,993 *(48,262)*	139,818 *(48,332)*	43 *(18)*	15.1 *(3.6)*	3.4 *(0.5)*	58 *(33)*	0.73 *(0.37)*	4.5

Filgrastim	4	8	22,595 *(6,076)*	175,323 *(50,665)*	176,145 *(51,004)*	32 *(8)*	14.7 (*3.9)*	2.8 *(0.6)*	42 *(19)*	0.54 *(0.27)*	5

AVI-014	8	8	31,460 *(7,927)*	312,569 *(81,029)*	313,525 *(81,043)*	35 *(11)*	11.0 *(2.2)*	3.7 *(0.3)*	35 *(18)*	0.45 *(0.23)*	5

Filgrastim	8	8	41,442 *(9,986)*	347,053 *(70,749)*	347,489 *(70,698)*	36 *(10)*	9.8 *(2.3)*	3.1 *(0.5)*	31 *(15)*	0.35 *(0.14)*	6

All values are means with (SD), except in the case of Tmax.

* = median

**Table 3:** Pharmacokinetic geometric mean ratios of AVI-014/filgrastim at 4 μg/kg

PK parameter	Dose mcg/kg	Geo Mean AVI-014 (n = 16)	Geo Mean Filgrastin (n = 8)	P value	Geo Mean Ratio (90% CI)
AUC_(0–72)_	4	169.545	170,259	0.9766	1.00 (0.76, 1.31)

Cmax	4	18,830	21,855	0.2952	0.86 (0.66, 1.13)

**Table 4:** Pharmacokinetic Geometric Mean ratios of AVI-014/Filgrastim at 8 μg/kg

**PK parameter**	**Dose μg/kg**	**Geometric Mean AVI-014 (n = 8)**	**Geometric Mean Filgrastin (n = 8)**	**P value**	**Geometric Mean Ratio (90% CI)**
AUC_(0–72)_	8	301,979	340,837	0.3669	0.89 (0.69, 1.14)

Cmax	8	30,467	40,325	0.0550	0.76 (0.58, 0.98)

### Pharmacodynamics

**Figure 4:**
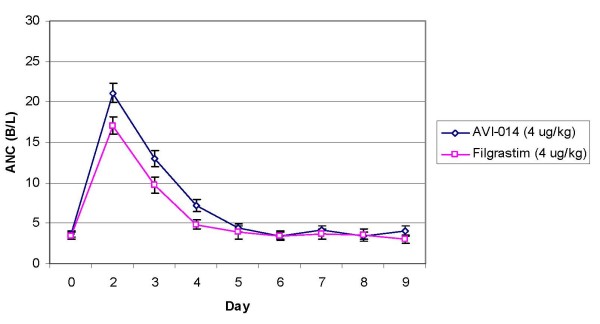
**4 μg/kg Dose of AVI-014 and Filgrastim Absolute Neutrophil Count Response, (with standard error)**.

**Figure 5:**
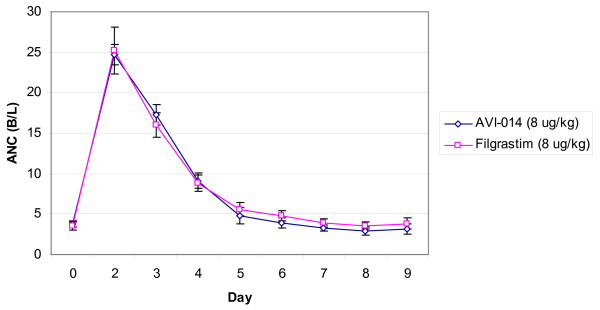
**8 μg/kg Dose of AVI-014 and Filgrastim Absolute Neutrophil Count Response, (with standard error)**.

Peak WBC and ANC occurred on day 2, with a return to baseline values at approximately day 6. CD 34+ peak values occurred on day 3 for the 4 μg/kg groups and day 4 for the 8 μg/kg groups. AVI-014 and filgrastim demonstrate similarity in WBC, ANC, and CD 34+ response at equal mass doses of drug. Geometric mean ratios of ANC, WBC and CD 34+ cell counts between treatments are presented in Tables 5, 6. Graphical representation of ANC is presented in Figures 4 and 5. Pharmacodynamic bioequivalence for WBC and ANC between AVI-014 and filgrastim was met, as 90% confidence intervals for the point estimate of the geometric mean ratios of AUC and Cmax lay within the a priori 0.5–2.0 bounds. The point estimates for CD 34+ of the geometric mean ratios were close to one, though the confidence intervals were high, reflecting the large variance seen in the data. As with pharmacokinetics, the geometric mean ratios between treatments did not appreciably change when analysis was performed using combined data from Panels A and B compared to using only blinded administration in Panel B.

**Table 5:** Pharmacodynamic geometric mean ratios of AVI-014/filgrastim at 4 μg/kg

**Hematologic**	**Parameter**	**Dose μg/kg**	**Geometric Mean AVI-014 (n = 8)**	**Geometric Mean Filgrastin (n = 8)**	**P value**	**Geometric Mean Ratio (90% CI)**
WBC	AUC_(0–9)_	4	1343.9	1193.6	0.1473	1.13 (0.98, 1.29)

	Cmax	4	375.1	326.7	0.1737	1.15 (0.97, 1.36)

ANC	AUC_(0–9)_	4	2146.3	1560.3	0.0489	1.38 (1.06, 1.78)

	Cmax	4	735.9	515.8	0.0690	1.43 (1.04, 1.96)

CD 34+	AUC_(0–6)_	4	3208.8	3088.9	0.9613	1.04 (0.27, 4.06)

	Cmax	4	1322.7	1186.3	0.8875	1.12 (0.29, 4.24)

**Table 6:** Pharmacodynamic geometric mean ratios of AVI-014/filgrastim at 8 μg/kg

**Hematologic**	**Parameter**	**Dose μg/kg**	**Geometric Mean AVI-014 (n = 8)**	**Geometric Mean Filgrastin (n = 8)**	**P value**	**Geometric Mean Ratio (90% CI)**
WBC	AUC_(0–9)_	8	1444.7	1574.1	0.3824	0.92 (0.78, 1.09)

	Cmax	8	415.9	435.7	0.6617	0.95 (0.79, 1.15)

ANC	AUC_(0–9)_	8	1974.4	2268.9	0.4626	0.87 (0.63, 1.21)

	Cmax	8	661.3	739.6	0.6230	0.89 (0.60, 1.34)

CD 34+	AUC_(0–6)_	8	3121.6	2791.2	0.8785	1.12 (0.31, 3.99)

	Cmax	8	1373.2	1315.9	0.9569	1.04 (0.26, 4.11)

### Safety results

#### Adverse events

All subjects completed the trial. There were no serious adverse events. A total of 47.5% of the enrolled subjects experienced at least one adverse event. Of a total of 42 adverse events, thirty-four events were judged as possibly related to study drug. The most common adverse events were headache, myalgia, back pain, and bone pain. 50.0% (12 of 24) of subjects treated with AVI-014 and 43.8% (7 of 16) of those treated with filgrastim had an adverse event. Musculoskeletal complaints, which included bone pain and muscle aches, occurred in 37.5% (9 of 24) subjects receiving AVI-014 and 18.8% (3 of 16) subjects receiving filgrastim. Headache occurred in 20.8% (5 of 24) subjects receiving AVI-014 and 18.8% (3 of 16) of subjects receiving filgrastim. A sensation of neck fullness was noted in two subjects on the day of drug administration. One subject noted headache, dizziness, chest discomfort, and nausea approximately 45 minutes after receiving AVI-014 4 μg/kg. This resolved and approximately eight hours later this subject noted a sensation of neck fullness. The event in this subject occurred 30 minutes after his roommate, who received filgrastim 4 μg/kg, noted neck fullness. Both episodes were self-limited, not associated with other signs of hypersensitivity, and considered possibly related to study drug. Injection site pain occurred in one subject receiving AVI-014.

#### Immunogenicity

For all subjects, serum neutralizing antibodies and binding antibodies were assessed by serum-mediated inhibition of in vitro cell proliferation assays and by ELISA detection/quantification of hα-G-CSF antibodies using goat α-human Ig-peroxidase conjugate (ZeptoMetrix). Blood sampling for immunogenicity testing was performed at pre-dose and on day 21 time points. No hG-CSF neutralizing antibodies were detected in pre-dose or day 21 samples from any subject. One patient who had received AVI-014 at 8 μg/ml showed low levels (<3 ng/ml) of anti-hG-CSF antibodies in both pre-dose and day 21 samples, with equivalent levels at both time points. Low levels of naturally occurring anti-G-CSF antibodies in normal subjects have previously been reported to be relatively common (11% of subjects) [6]. Thus, no treatment-related immunogenicity was observed.

## Discussion

This study describes the first human administration of AVI-014, an avian-derived, human, glycosylated G-CSF. AVI-014 was well tolerated, with a side effect profile of myalgia and bone pain that was comparable to that seen with a similar dose of filgrastim. The product insert for filgrastim notes a ~3.5 hour elimination half life [7,8], which corresponds to the estimated alpha half life noted in the present study. Filgrastim has been previously described in a two compartment model with dose-dependent, saturable elimination [9]. We employed a sensitive G-CSF assay with a limit of quantification of 7.8 pg/ml, which is an order of magnitude more sensitive than those employed by Wang in 2001 [9] or Borleffs in 1998 [8]. The capture of data points closer to the limit of quantification of the assay allowed for the detection of the terminal elimination phase at the doses administered in the trial. This also accounts for differing values of volume of distribution compared to the product insert and earlier published information. Other pharmacokinetic parameters observed for filgrastim are similar to those previously published [7,10].

The comparative extent of G-CSF exposure, as determined by AVI-014 and filgrastim AUC(0–72) values, appeared comparable at 4 μg/kg and 8 μg/kg dose. However, the 90% confidence interval of the geometric mean ratio is outside a priori bioequivalence bounds of 0.8–1.25. It should be noted that this study employed a parallel and not crossover design and thus does not constitute a formal exploration of bioequivalence. A parallel design was chosen because of unknown immunogenicity of AVI-014 in this first-in-human clinical trial. There was no evidence of immunogenicity noted in this trial, which is consistent with the experience of an egg white-derived interferon alpha-2b [11]. The use of a parallel and not crossover design is not expected to bias the analysis toward bioequivalence. Indeed, intra-subject variability introduced by the parallel design employed in the present study would likely make demonstration of bioequivalence more difficult.

The response of each product in terms of WBC, neutrophil response and CD 34+ was similar between treatments. This is consistent with previous observations which demonstrated a close linkage between the serum concentration of recombinant G-CSF and neutrophil response [12], as well as other observations suggesting similarity of response to glycosylated and non-glycosylated recombinant G-CSF preparations [13]. Pharmacodynamic bioequivalence for WBC and ANC between AVI-014 and filgrastim was met, as 90% confidence intervals for the geometric mean ratios of AUC and Cmax lay within the a priori 0.5–2.0 bounds. The geometric mean ratios for CD 34+ were close to 1, though the confidence intervals were high possibly due to the large variance seen in the data.

## Conclusion

In conclusion, AVI-014 was well tolerated in healthy subjects with no evidence of immunogenicity. AVI-014 has a pharmacokinetic profile in healthy volunteers that is comparable to that of filgrastim and generates a pharmacodynamic response in terms of neutrophil mobilization that is equivalent to that of filgrastim. The present report supports further investigations of AVI-014 in patient populations.

## Abbreviations

CI: confidence interval; ECG: electrocardiogram; G-CSF: granulocyte colony-stimulating factor; rhG-CSF: recombinant human granulocyte colony stimulating factor; GMR: Geometric Mean Ratio

## Competing interests

This study was funded by Synageva Biopharma (formerly known as AviGenics, Inc.). Dr. Varki was supported by NIH Postdoctoral training grant T32 GM08562. Dr. Kraft has received research grants from, and is a paid consultant to, Synageva Biopharma.

## Authors' contributions

RV prepared the first draft of the manuscript, edited revisions, and assisted in the conduct of the clinical trial. EP performed statistical analysis. MCL performed analytical analysis, edited the manuscript drafts and revisions, and contributed to the study design. AF assisted with study design and conduct of the clinical trial. WKK designed study protocol, performed pharmacokinetic analysis, revised and edited first draft of manuscript, prepared revised drafts of the manuscript, and was responsible for overall conduct of the clinical trial. All authors read and approved the final manuscript.

## Pre-publication history

The pre-publication history for this paper can be accessed here:


